# A New Method for Computing Attention Network Scores and Relationships between Attention Networks

**DOI:** 10.1371/journal.pone.0089733

**Published:** 2014-03-03

**Authors:** Yi-Feng Wang, Qian Cui, Feng Liu, Ya-Jun Huo, Feng-Mei Lu, Heng Chen, Hua-Fu Chen

**Affiliations:** 1 Key Laboratory for NeuroInformation of Ministry of Education, University of Electronic Science and Technology of China, Chengdu, Sichuan, People's Republic of China; 2 School of Life Science and Technology, University of Electronic Science and Technology of China, Chengdu, Sichuan, People's Republic of China; 3 School of Political Science and Public Administration, University of Electronic Science and Technology of China, Chengdu, Sichuan, People's Republic of China; Centre de Neuroscience Cognitive, France

## Abstract

The attention network test (ANT) is a reliable tool to detect the efficiency of alerting, orienting, and executive control networks. However, studies using the ANT obtained inconsistent relationships between attention networks due to two reasons: on the one hand, the inter-network relationships of attention subsystems were far from clear; on the other hand, ANT scores in previous studies were disturbed by possible inter-network interactions. Here we proposed a new computing method by dissecting cue-target conditions to estimate ANT scores and relationships between attention networks as pure as possible. The method was tested in 36 participants. Comparing to the original method, the new method showed a larger alerting score and a smaller executive control score, and revealed interactions between alerting and executive control and between orienting and executive control. More interestingly, the new method revealed unidirectional influences from alerting to executive control and from executive control to orienting. These findings provided useful information for better understanding attention networks and their relationships in the ANT. Finally, the relationships of attention networks should be considered with more experimental paradigms and techniques.

## Introduction

Attention plays important roles in every aspect of human behavior, ranging from basic perception to complex cognition and emotion. It is widely accepted that attention can be divided into at least three separate subsystems: the alerting, orienting, and executive control networks [1]–[3]. Alerting is to achieve and maintain a state of high sensitivity to incoming stimuli; orienting refers to the selection of information from sensory input; and executive control is defined as involving the mechanisms for resolving various conflicts [4].

To test the efficiency and independence of the three attention networks, Fan and colleagues [1] devised an attention network test (ANT). The ANT combines Posner's cued reaction time (RT) task [5] and Eriksens' flanker task [6] to differentiate independent attention components in one paradigm. This paradigm consistently induced remarkable main effects of attention networks and has been demonstrated to be reliable between sessions [1], [7], [8]. In the past decade, it has been widely employed in brain functional [9]–[12], developmental [8], [13], [14], genetic [15]–[17], and psychiatric investigations [18]–[23] to test normal and abnormal attention abilities.

However, the interactions between attention networks are complicated and inconsistent. Table 1 showed the results of 14 visual attention researches using the ANT in normal adults. Strong interactions between attention networks appeared in only half of these studies [9], [11], [24]–[28]. Some studies observed weak interactions [1], [29], but others failed to find any interactions [10], [13], [30], [31].

**Table 1:** Main effects of and interactions between attention networks in attention network test (ANT).

		Main effects	Interactions
Publication	Paradigm	AL	OR	EX	AL×OR	AL×EX	OR×EX
Fan et al.2002	S-ANT	s	s	s	ns	s	ns
Callejas et al.2004	D-ANT	s	s	s	s	s	s
Callejas et al.2005	D-ANT	s	s	s	s (100 ms)	s	s
Fan et al.2005	S-ANT	s	s	s	ns	ns	ns
Konrad et al.2005	S-ANT	s	s	s	ns	ns	ns
Roberts et al.2006	D-ANT	V∶s	V∶s	V∶s	-	-	-
	D-ANT	A∶s	A∶ns	A∶s	-	-	-
Fan et al.2007	S-ANT	s	s	s	-	s	s
Fuentes et al.2008	D-ANT	s	s	s	s (<800 ms)	-	-
Ishigami et al.2009	S-ANT	s	s	s	ns	s	ns
	D-ANT	s	s	s	s	s	s
Fan et al.2009	S-ANT	s	s	s	-	s	s
Ishigami et al.2009	S-ANT	s	s	s	-	s	ns
	D-ANT	s	s	s	ns	s	s
Kellermann et al.2011	S-ANT	s	s	s	-	s	s
McConnell et al.2011	S-ANT	s	s	s	-	s	s
Liu et al.2013	S-ANT	s	s	s	ns	ns	ns

Note: AL, alerting; OR, orienting; EX, executive control; S-ANT, single-modality ANT; D-ANT, double-modality ANT; V, visual; A, auditory; s, significant; ns, non-significant; “-”, no testing.

Unstable interactions between attention networks might be due to several variables, such as modality, stimulus onset asynchrony (SOA), and inhibition of return (IOR). Roberts and colleagues [31] provided direct evidence that modality impacts on the efficiency of attention systems, especially on the orienting network. Ishigami and Klein [28], [29] reported that the double-modality ANT increases the interaction between orienting network and the other two networks. These results may be affected by two reasons: first, different mechanisms under visual attention and auditory attention, or sensory integration may alter the processing of attention networks; second, the alerting signal appears twice in successive alerting and orienting cues, because the spatial cue inherently includes temporal and spatial information.

The SOA is reported to be associated with the efficiency of alerting network. Several studies showed that the alerting effect lasts no longer than 900 ms and peaks at around 400 ms [11], [24], [25], [32], [33]. They proposed that alerting effect was related to alpha band suppression peaked at about 400 ms [11] and increased the general alerting level during a limited time window [32].

The validity of spatial cue is used to test a famous phenomenon called inhibition of return [34], [35], which refers to a mechanism that encourages orienting towards novel locations in cue–target onset asynchronies (CTOA) longer than 200 ms. However, the IOR disappeared in ANT studies [26], [32], [36]. Whether and how this phenomenon involves in orienting/reorienting processing and intervenes between orienting network and other networks need further clarification.

Although some studies using the ANT failed to reveal interactions of attention networks and some others reported interactions which are contaminated by various factors, evidence from genetic and neuroimaging researches indicated possible dependence of attention networks. For example, the *monoamine oxidase a* (MAOA) gene and *catechol-o-methyl transferase* (COMT) gene were observed to be associated with both alerting network [37] and executive control network [15], [38], [39], while the *apolipoprotein E* (APOE) gene was associated with both orienting network [40], [41] and executive control network [42]. Neuroimaging studies have confirmed that attention networks involve overlapping areas of brain activity [4]. First, lateral and medial areas of frontal lobe, such as the anterior cingulate cortex (ACC), ventral- and dorso-lateral prefrontal cortex (VLPFC/DLPFC), and insula, participated in the processing of alerting [3], [43], [44] and executive control [45], [46]. Second, the temporal–parietal junction (TPJ) was involved in alerting [21] and orienting [47], [48]. Third, the cerebellum is important for orienting [49] and executive control [50]. Briefly, genetic, neuroimaging, and behavioral studies using the ANT and other paradigms suggested the possibility of dependence of attention networks.

If the inter-network relationships were striking, the traditional computing method of ANT scores would be affected by inter-network interactions. For instance, the Equation (10) for computing conflict effect contains conditions of center cue and spatial cue, thus might be influenced by alerting and orienting networks. Therefore, we introduced a new computing method to re-analyze efficiencies of attention networks and their relationships. Before computing ANT scores, we dissected six conditions into different components:

*No-cue congruent (ncc): baseline*

*No-cue incongruent (nci): baseline + executive control*

*Center-cue congruent (ccc): baseline + alerting*

*Center-cue incongruent (cci): baseline + alerting + executive control*

*Spatial-cue congruent (scc): baseline + alerting + orienting*

*Spatial-cue incongruent (sci): baseline + alerting + orienting + executive control*

Based on the above dissection, we computed ANT scores and inter-network relationships as follows:
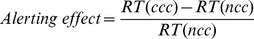
(1)

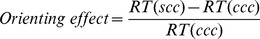
(2)

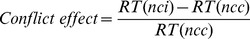
(3)

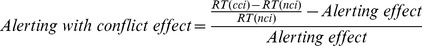
(4)

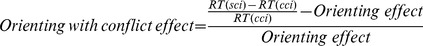
(5)

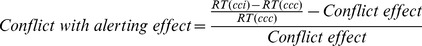
(6)

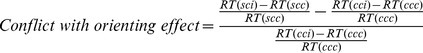
(7)


The first three equations computed scores of alerting, orienting, and executive control networks, respectively. The last four equations estimated influences of executive control network to alerting network and to orienting network, and influences of alerting network and orienting network to executive control network, respectively. In order to avoid the baseline difference on different conditions and to compare between two computing methods, the relevant baseline RT was adopted for both old [51] and new computing methods. All RTs in equations of this paper refer to the median of RTs of all trials in each condition. Equations for ANT scores with the old method were as follows:
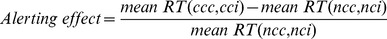
(8)

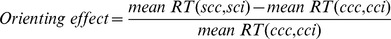
(9)

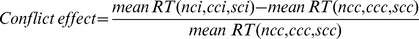
(10)


Our aims in the current study were (1) to examine ANT scores and inter-network relationships with the new computing method, and (2) to compare the efficiency of the two methods. Based on previous evidence, we hypothesized that ANT scores would show some differences between the two methods, and the new method would reveal correlations and interactions between attention networks.

## Methods

### Subjects

Thirty nine graduates and undergraduates participated in the experiment. Three of them were excluded due to too low accuracy (<75%) in any of the six conditions. The remaining participants (19 women; ages from 19 to 28, mean age = 23.54, standard deviation (SD) = 1.85) were right handed (selected by the Chinese version of Edinburgh-Handedness Questionnaire, coefficients>60) with correct to normal vision. All of them had no history of neurological disorder and major psychiatric disorder.

### Ethics Statement

This study was approved by the research ethical committee of School of Life Science and Technology, University of Electronic Science and Technology of China, and written informed consent was obtained from each subject.

### Procedure

**Figure 1:**
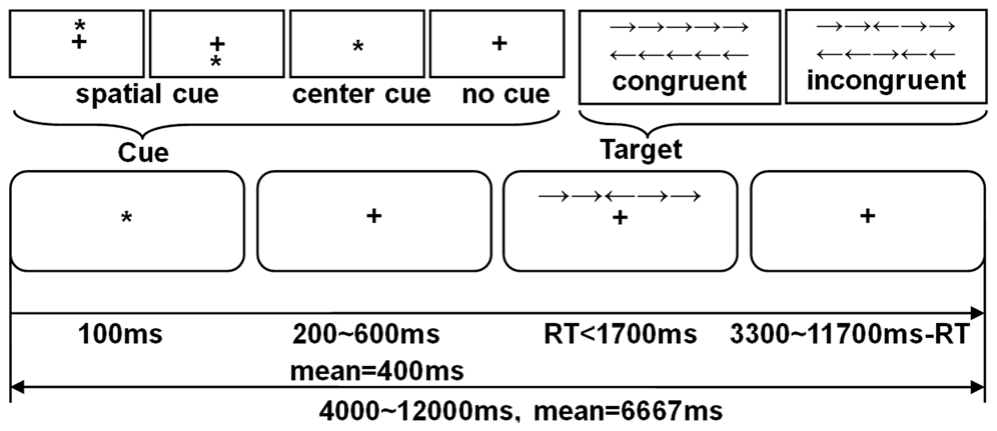
Schematic of the condensed version of attention network test.

A condensed version of attention network test (ANT) with three cue conditions (no cue, center cue, spatial cue) and two target conditions (congruent and incongruent) was used. At the beginning of a trial, a cue (none, center, or spatial cue) appeared for 100 ms. After a variable duration (200∼600 ms), the target (the center arrow) and flankers (congruent or incongruent) were simultaneously presented until the participant responded with a button press, but for no longer than 1700 ms. After that, the target and flankers disappeared immediately and a post-target fixation period lasted for a variable duration. A single trial lasted for 4000∼12000 ms (see Figure 1).

The experiment consisted of 3 blocks of 54 trials (3 cue conditions ×2 target conditions ×9 time intervals) with no feedback. Cue×target conditions were counterbalanced in each block. Participants were asked to focus on a fixation located on the center of the screen throughout the experiment, and to respond as quickly and accurately as possible. Before the experiment, a practice procedure of about 3 min was performed.

Stimuli were presented via E-Prime 2.0 (http://www.pstnet.com; Psychology Software Tools, Inc), on a 14-in Dell laptop running Windows 7 operational system. The distance from participants' eyes to the screen was about 60 cm. Visual angles were set according to Fan et al. [10]'s study. Responses were collected via Q (for left targets) and P (for right targets) on the keyboard.

### Analysis

To remove outliers, all RTs <200 ms and >1200 ms in each condition were removed (1.53% data). The accuracy analysis was based on the remaining data. Further, the RT analysis was based on correct responses. Since RTs were not normally distributed, we used median RT of each condition as raw scores for each subject [51]. Thus, median was used when talking about RT for one condition or for one subject both in equations and other parts of this paper.

We first calculated each score for every subject using Equations (1)–(10), and assessed the effect of these scores using one sample *t*-tests. Three old ANT scores and three new ANT scores were then compared using paired-samples *t*-tests to examine their differences.

Second, bivariate correlation (Pearson's *r*) was used to examine the statistical independence of attention networks. Accuracy data were not recruited for correlation analysis, because some differences of accuracy between two conditions were zero which would make invalid denominators in the equations for ANT scores.

Third, repeated measures ANOVAs were adopted to explore the efficiency and independence of attention networks. For the old method, 3 (cue)×2 (target) ANOVAs were run for accuracy and RT, respectively. This would provide information of main effects and interactions of the cue and target. For the new method, data of both accuracy and RT were divided into two parts: (I) alerting vs. executive control (including ncc, nci, ccc, and cci conditions); (II) orienting vs. executive control (including ccc, cci, scc, andsci conditions). The segmentation allows us to test the effect of each ANT score and interactions between ANT scores. All Post hoc analyses were based on paired-samples *t*-tests. Greenhouse-Geisser method was used to adjust effects of *F*-test wherever the spherical assumption had been violated.
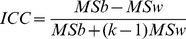
(11)


To further examine the reliability of both the old and new computing methods, we run the intra-class correlation (ICC) across three runs and split-half reliability tests. The ICC is a common index of measuring test–retest reliability [52]. Using a one-way ANOVA with random subject effects, we splited the total sum of the mean squares into between-subject (MSb) and within-subject (MSw, e.g., residual error) sum of mean squares. ICC values were calculated according to Equation (11) where *k* represents the number of repeated observations per participant. The ICC was analyzed in the individual level with one subject having one value for one item (e.g., the new alerting score) in each run. There were three items for the old ANT score and the new ANT score (alerting, orienting, and executive control), and four items for the relationship between attention networks (alerting with conflict, orienting with conflict, conflict with alerting, and conflict with orienting). Further, the split-half reliability was computed using the method proposed by Ishigami and Klein [8]. That is, using a permutation method in the individual level, all ANT scores or relationship scores between attention networks were randomly split into two halves 1000 times (less than Ishigami and Klein's permutation of 10000 times). A correlation was calculated for each split, and reliability was the mean of 1000 correlation coefficients. Noting that only RT data were used for ICC and split-half analyses, because 1) some differences of accuracy between two conditions were zero which would make invalid denominators in the equations for ANT scores and 2) the relationship between attention networks cannot be measured using zero ANT scores.

## Results

**Figure 2:**
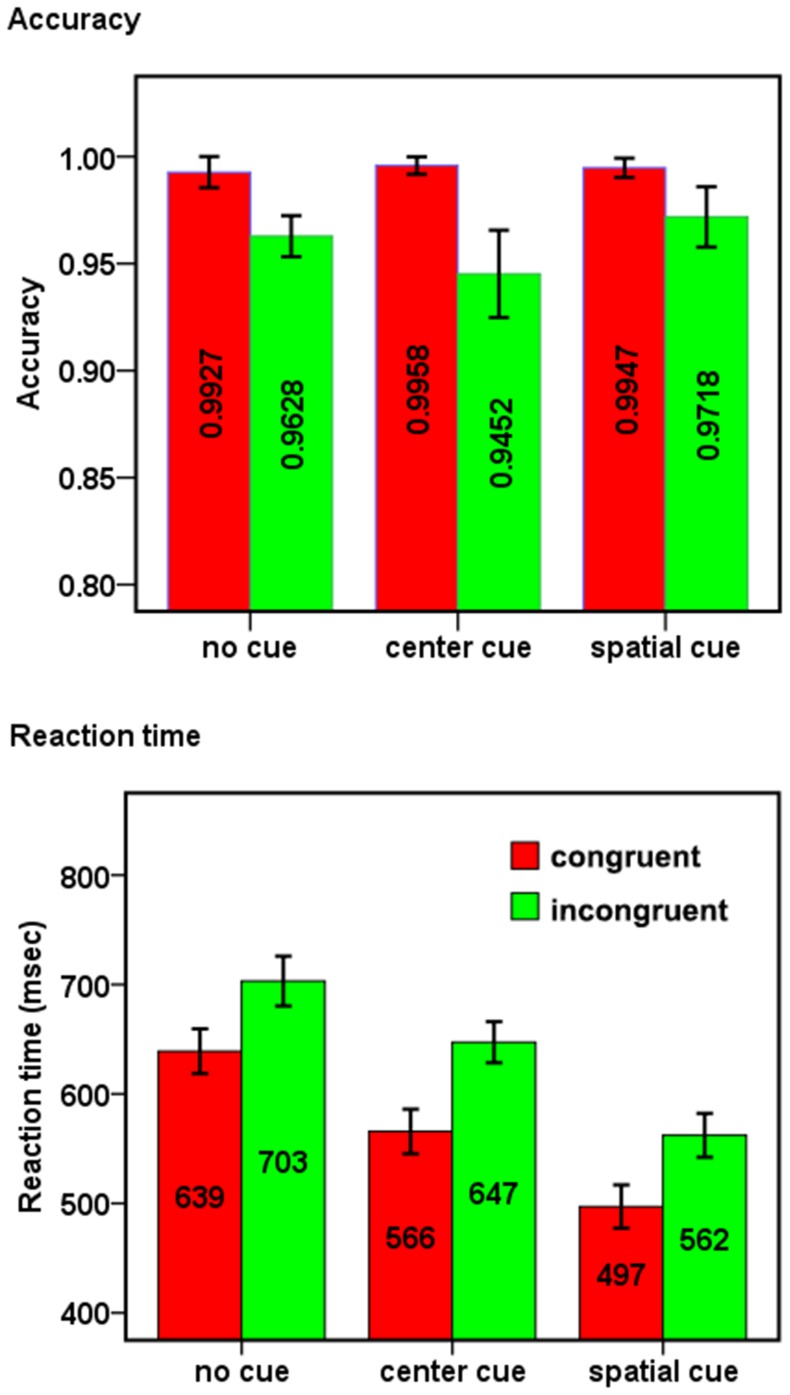
The results of descriptive statistics. The (a) accuracy and (b) reaction time of each condition. Error bars showed the 95% confidence interval.

**Figure 3:**
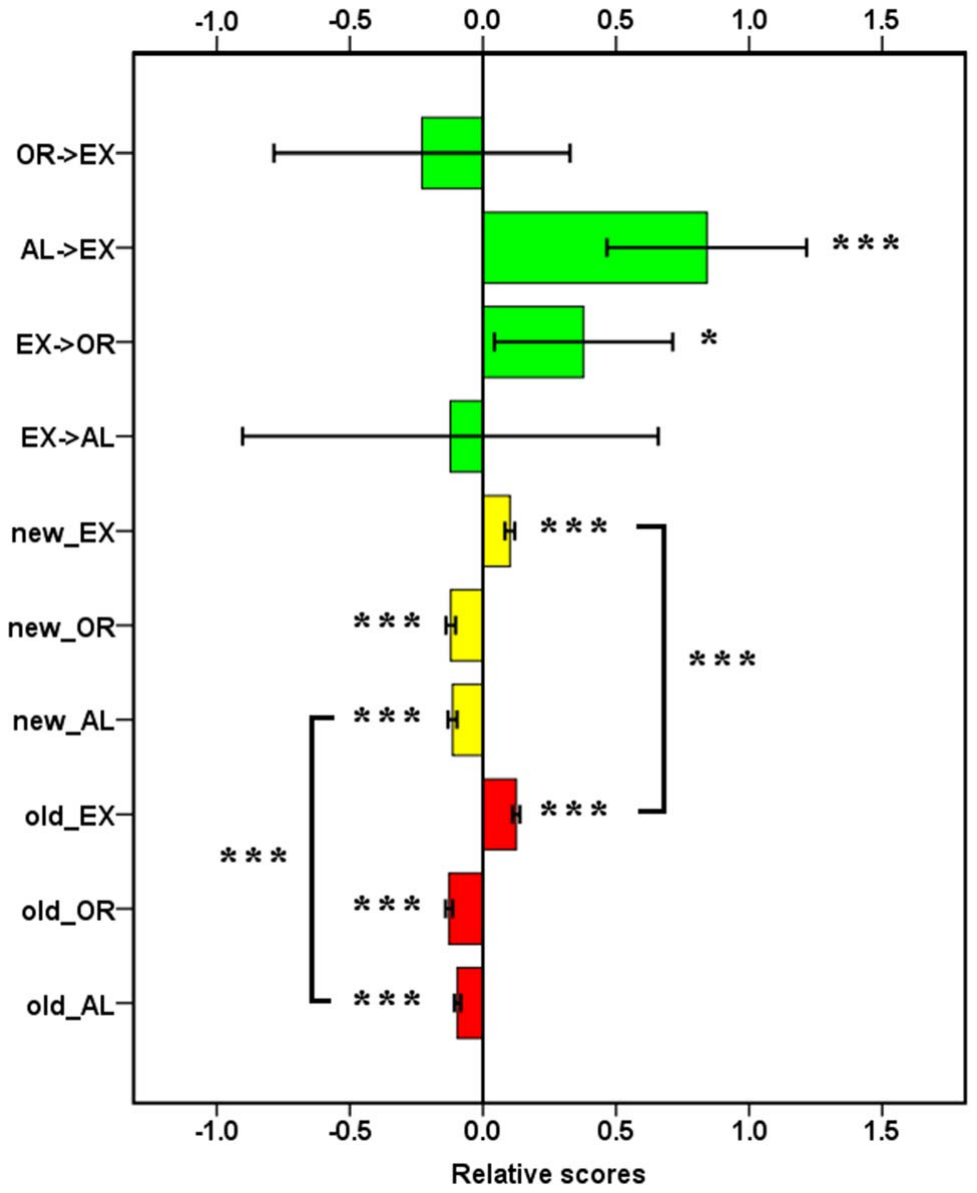
ANT scores and relationships between alerting network and executive control network, and between orienting network and executive control network. Error bars showed the 95% confidence interval. AL: alerting; EX: executive control; OR: orienting; ->: to; *: *p*<0.05; ***, *p*<0.001.

Figure 2 shows accuracy and RT under each condition. Based on these data, we calculated ANT scores and inter-network relationships (Figure 3) with Equations (1)–(10).

### ANT scores and relationships of attention networks

For ANT scores with the old method, one sample *t*-test showed that effects of alerting [*t* (35) = 15.62, *p*<0.001], orienting [*t* (35) = 19.27, *p*<0.001], and executive control [*t* (35) = 18.31, *p*<0.001] were all significant. This result is in line with most of previous findings. For the scores with the new method, effects of alerting [*t* (35) = 13.50, *p*<0.001], orienting [*t* (35) = 13.79, *p*<0.001], and executive control [*t* (35) = 11.15, *p*<0.001] were also significant. We further compared scores of alerting, orienting and executive control by paired-samples *t*-test. Results showed that the old method computed a smaller alerting score [*t* (35) = 3.73, *p* = 0.001] and a larger executive control score [*t* (35) = 3.55, *p* = 0.001] than the new method did. The orienting score was equivalent [*t* (35) = 1.26, *p* = 0.216] between the two methods. This indicated that the ANT was a credible paradigm to detect three attention networks. However, scores with the old method might be contaminated by inter-network interactions.

The relationships of attention networks were computed with Equations (4)–(7) and estimated by one sample *t*-test (see Figure 3). Results showed unidirectional influence from alerting network to executive control network [*t* (35) = 4.56, *p*<0.001] and from executive control network to orienting network [*t* (35) = 2.29, *p* = 0.03]. Specifically, alerting increased the gap between congruent and incongruent conditions and executive control increased the difference between spatial cue and center cue conditions. These results were similar to inter-network interactions reported in Fan and colleagues' original article [1]. However, executive control did not influence alerting [*t* (35) = −0.32, *p* = 0.75]. Orienting also exerted no influence on executive control [*t* (35) = −0.84, *p* = 0.41]. These findings provided evidence for the dependence between attention networks.

### Correlations between attention networks

In order to verify the dependence between attention networks, we performed Pearson correlations on both old and new computing methods. For the old method, correlation coefficients of RTs of three networks were −0.13 (alerting vs. executive control; *p* = 0.463), 0.03 (alerting vs. orienting; *p* = 0.865), and 0.14 (executive control vs. orienting; *p* = 0.406). For the new method, correlation coefficients were 0.30 (alerting vs. executive control; *p* = 0.061), −0.01 (alerting vs. orienting; *p* = 0.974), and 0.44 (executive control vs. orienting; *p* = 0.007). In keeping with the unidirectional influence from alerting to executive control to orienting, the current findings revealed that both correlations between alerting and executive control and between executive control and orienting approached (marginal) significance, thus providing further evidence for their dependence.

### ANOVA for the old method

For the accuracy, a remarkable main effect of cue was observed, *F* (2, 70) = 3.66, *p* = 0.039, partial *η* ^2^ = 0.095. The accuracy of center cue condition is lower than that of spatial cue condition (*p* = 0.013). The main effect of target also approached significance, *F* (1, 35) = 42.64, *p*<0.001, partial *η* ^2^ = 0.549. The accuracy of congruent condition is higher than that ofincongruent one. The interaction of cue by target was also significant: *F* (2, 70) = 4.19, *P* = 0.024, partial *η* ^2^ = 0.107. Post hoc *t*-test showed that the effect of cue appeared only on the incongruent condition (*t* (35) = 3.06, *p* = 0.004 for center cue condition vs. spatial cue condition).

For the RT, both effects of cue [*F* (2, 70) = 387.80, *p*<0.001, partial *η* ^2^ = 0.917] and target [*F* (1, 35) = 388.55, *p*<0.001, partial *η* ^2^ = 0.917] were significant. The RTs of no cue, center cue, spatial cue conditions decreased successively (*ps*<0.001). The RT of congruent condition was shorter than that of incongruent one. The interaction of cue by target was also significant: *F* (2,70) = 4.11, *p* = 0.02, partial *η* ^2^ = 0.105. The difference between congruent conditon and incongruent condition was smaller on no cue condition [*t* (35) = 11.48, *p*<0.001] than that on center cue condition [*t* (35) = 14.02, *p*<0.001] and on spatial cue condition [*t* (35) = 14.95, *p*<0.001].

### ANOVA for the new method

As shown in the Methods section, data of accuracy and RT were divided into two parts: (I) alerting vs. executive control and (II) orienting vs. executive control.

(I) The RT analysis showed remarkable effects of alerting [*F* (1, 35) = 200.25, *p*<0.001, partial *η* ^2^ = 0.851] and executive control [*F* (1, 35) = 257.87, *p*<0.001, partial *η* ^2^ = 0.88]. The interaction of alerting by executive control was also significant: *F* (1, 35) = 6.25, *p* = 0.017, partial *η* ^2^ = 0.151. Alerting slightly increased the effect of executive control [*t* (35) = 4.95, *p*<0.001 vs. *t* (35) = 5.44, *p*<0.001].

Analysis of accuracy revealed a significant effect of executive control: *F* (1, 35) = 47.59, *p*<0.001, partial *η* ^2^ = 0.576. The accuracy of congruent condition was higher than that of incongruent one. The main effect of alerting [*F* (1, 35) = 1.63, *p* = 0.21, partial *η* ^2^ = 0.045] and interaction of alerting by executive control [*F* (1, 35) = 3.21, *p* = 0.082, partial *η* ^2^ = 0.084] were far from significant.

(II) The RT analysis showed remarkable main effects of orienting [*F* (1, 35) = 349.24, *p*<0.001, partial *η* ^2^ = 0.909] and executive control [*F* (1, 35) = 337.75, *p*<0.001, partial *η* ^2^ = 0.906]. A significant interaction between orienting and executive control could also be found: *F* (1, 35) = 6.37, *p* = 0.016, partial *η* ^2^ = 0.154. Incongruence increased the effect of orienting slightly [*t* (35) = 16.82, *p*<0.001 vs. *t* (35) = 12.72, *p*<0.001].

For the accuracy, we observed significant main effects of orienting [*F* (1, 35) = 9.38, *p* = 0.004, partial *η* ^2^ = 0.211] and executive control [*F* (1, 35) = 25.49, *p*<0.001, partial *η* ^2^ = 0.421]. There was also an interaction between orienting and executive control: *F* (1, 35) = 8.25, *p* = 0.007, partial *η* ^2^ = 0.191. Incongruence rather than congruence [*t* (35) = 3.06, *p* = 0.004 vs. *t* (35) = 0.45, *p* = 0.655] increased the effect of orienting.

### Reliability of the old and new methods

The ICC value of the new ANT scores (0.512) was much higher than that of the old ANT scores (0.035) and the relationship between the attention networks (0.194), indicating the high test–retest reliability of the new method. Similarly, the split-half reliability revealed that the new ANT scores (0.712) had higher internal consistency than the old ANT scores (0.039) and the relationship of ANT (0.263) which confirmed the reliability of the new ANT scores.

## Discussion

In order to reduce the interference from inter-network interactions in computing ANT scores and estimate specific relationships between attention networks, we dissected six ANT conditions in detail. According to the dissection, we put forward a new method with 7 equations to compute ANT scores and relationships between attention networks. This method computed relative pure ANT scores and unidirectional relationships between attention networks except for main effects and interactions of attention networks.

### ANT scores

Comparing to the old method, the new method computed a larger alerting score and a smaller executive control score. This difference might be due to the interplay of alerting network and executive control network. As suggested by Callejas et al. [24], [25] and Fan et al. [26], the alerting network could increase the score of executive control network. Our results (Figure 3) also showed that alerting network increased executive control score, whereas executive control network decreased alerting score. For this reason, the old method underrated alerting score and overvalued executive control score. This difference between results of two methods indicated the dependence of alerting network and executive control network and the necessity of modifying computing method of ANT scores.

Both old and new methods revealed significant efficiency of ANT scores. This added weight to the reliability of the ANT in testing the efficiency of attention networks [1], [7], [28]. Furthermore, ANOVA showed significant main effects of alerting, orienting, and executive control networks. Considering not all studies reported significant effects of attention networks [7], ANT scores, especially that scores with the new method, might be preferable in testing the efficiency of attention networks.

### Relationships between attention networks

Results from inter-network relationships computed with the new method, correlation analysis, and ANOVA consistently revealed the dependence of attention networks.

Firstly, the new method revealed a unidirectional influence from alerting network to executive control network and from executive control network to orienting network. This finding confirmed the dependence between attention networks [8], [11], [24], [25], [28], [32], [33], [53]. Similar to Callejas et al.'s [24], [25] studies using double-modality paradigm and Fan et al. [26]'s study using single-modality paradigm, our results showed an inhibition from alerting network to executive function network. It suggested that this influence was modality independent. However, the relationship between orienting and executive control was opposite to Callejas et al.'s finding. There may be three reasons for this inconsistency. The first is the instability of inter-network relationships. Figure 3 showed much larger standard deviations of inter-network relationships than ANT scores. The large variation may result in the absence of interactions of attention networks in some studies [1], [10], [13] and the lack of influence from orienting network to executive control network in our study. Another reason is the alerting signal appeared twice in successive alerting and orienting cues in Callejas et al.'s studies. Thus, the influence from orienting to executive control was possibly impacted by successive alerting signals. The third reason involves other potential variables such as modality, SOA, and attention state. These variables could participate in inter-network interactions, as we discussed in the introduction section. Although the current results (Figure 3) showed some clues of reciprocal relationships between alerting network and executive control network and between executive control network and orienting network, credible inter-network relationships are far from clear.

Secondly, the new method rather than the old method uncovered correlations between alerting and executive control and between executive control and orienting. Previously, some studies with the old method failed to find any correlations between attention networks [1], [10], [11], whereas some others found striking inter-network correlations between alerting RT and orienting RT, between alerting accuracy and executive accuracy, between orienting accuracy and executive accuracy, and between orienting RT and executive accuracy [7]. For instance, Fossella et al. [15] observed a negative correlation between alerting and executive control with a large sample (200 adults). Recently, Westlye et al. [51] observed negative correlations between orienting and alerting and between orienting and executive control. We suggested that the absence of correlations in some studies may be caused by the old ANT scores. According to evidence for interactions and overlapping genes and brain structures between attention networks (see the introduction section), the emergence of correlations should be reasonable.

Thirdly, significant interaction between cue and target with the old method and interaction between alerting and executive control and between executive control and orienting with the new method were found. Significant interactions between attention networks were also found in previous studies [9], [11], [26]. Posner [54] proposed the alerting network produces an inhibitory effect on the executive function network to enhance fast responses to sensory input and prevent the system from focusing on feelings or thoughts or on further processing of the stimulus. For the relationship between orienting and executive control, some researches [1], [25] suggested the spatial cue allows the participant to concentrate on this area and ignore the incongruent flankers. These explanations focused on unidirectional influences from alerting to executive control and from orienting to executive control; nevertheless, influences from executive control to alerting and to orienting have never been concerned in previous correlation and interaction analyses. Although with instability, our results showed the possibility of bidirectional influences. As mentioned by Callejaset al. [24]: even though the functions and neural substrates of three attention networks are distinct, they can act under the constant influence of each other in order to produce an efficient and adaptive behavior.

### Merits and limitations

A new method for calculating ANT scores and their relationships was proposed with remarkable advantages compared to the old method. First, the new ANT scores may eliminate impacts from inter-network interactions more successfully than the old ANT scores; second, the new method is demonstrated to be more reliable than the old method; third, the new method allows us to directly estimate the inter-network influences. Considering the widely usage of the ANT in studying genetic and neural mechanisms of attention networks [2], [55] and in investigating the abnormal attention networks in various diseases [19], [21]–[23], [30], [53], more pure and reliable markers are of vital importance for better estimating attention networks.

However, there are some limitations in our study. First, the unidirectional influences need further verification in a large sample, because of the instability of inter-network relationships. Whether applying the new method to published data would produce the same results also needs inspection. Second, the new computing method could not extract pure ANT scores, because of the existence of inter-network interactions in the ANT. To avoid cross impacts, future studies should test efficiency of attention networks in separate blocks. Third, we did not adopt the double cue condition. The no cue-double cue and the no cue-center cue may have about the same discrepancy scores [1], but whether they affect the inter-network interaction need further clarification. We suggest that all cccs and ccis in our equations could be replaced with dccs (double cue congruents) and dcis (double cue incrongurents). The discrepancies between equations with ccc/cci and those with dcc/dci should be assessed in future studies. It is worth noting that individual scores correlation with physiological/neuroimaging markers may be the best way to show that these new scores better describe the attention networks, which should be tested in future investigations. Last, the traditional single-modality ANT should be amended to improve the capacity for testing attention networks, given the absence of main effects of attention networks in some ANT studies [7] and inconsistent brain activities for each attention network [9], [10], [13], [30].

### Conclusion

We improved the method for computing ANT scores and relationships between attention networks. The new method could estimate more pure ANT scores and more specific inter-network relationships than the traditional method. It revealed a larger alerting score but a smaller executive control score than the old method. Further, significant correlations and interactions between alerting and executive control and between executive control and orienting were observed with data computed by the new method. Overall, we support that the ANT is a reliable paradigm to test the efficiency of attention networks rather than their interactions.
